# Task Shifting for Scale-up of HIV Care: Evaluation of Nurse-Centered Antiretroviral Treatment at Rural Health Centers in Rwanda

**DOI:** 10.1371/journal.pmed.1000163

**Published:** 2009-10-13

**Authors:** Fabienne Shumbusho, Johan van Griensven, David Lowrance, Innocent Turate, Mark A. Weaver, Jessica Price, Agnes Binagwaho

**Affiliations:** 1 Family Health International, Kigali, Rwanda; 2 TRACPlus - Center for Infectious Disease Control, Kigali, Rwanda; 3 Family Health International, Durham, North Carolina, United States of America; 4 Rwanda National AIDS Control Commission, Kigali, Rwanda; Boston University, United States of America

## Abstract

**Background:**

The shortage of human resources for health, and in particular physicians, is one of the major barriers to achieve universal access to HIV care and treatment. In September 2005, a pilot program of nurse-centered antiretroviral treatment (ART) prescription was launched in three rural primary health centers in Rwanda. We retrospectively evaluated the feasibility and effectiveness of this task-shifting model using descriptive data.

**Methods and Findings:**

Medical records of 1,076 patients enrolled in HIV care and treatment services from September 2005 to March 2008 were reviewed to assess: (i) compliance with national guidelines for ART eligibility and prescription, and patient monitoring and (ii) key outcomes, such as retention, body weight, and CD4 cell count change at 6, 12, 18, and 24 mo after ART initiation. Of these, no ineligible patients were started on ART and only one patient received an inappropriate ART prescription. Of the 435 patients who initiated ART, the vast majority had adherence and side effects assessed at each clinic visit (89% and 84%, respectively). By March 2008, 390 (90%) patients were alive on ART, 29 (7%) had died, one (<1%) was lost to follow-up, and none had stopped treatment. Patient retention was about 92% by 12 mo and 91% by 24 mo. Depending on initial stage of disease, mean CD4 cell count increased between 97 and 128 cells/µl in the first 6 mo after treatment initiation and between 79 and 129 cells/µl from 6 to 24 mo of treatment. Mean weight increased significantly in the first 6 mo, between 1.8 and 4.3 kg, with no significant increases from 6 to 24 mo.

**Conclusions:**

Patient outcomes in our pilot program compared favorably with other ART cohorts in sub-Saharan Africa and with those from a recent evaluation of the national ART program in Rwanda. These findings suggest that nurses can effectively and safely prescribe ART when given adequate training, mentoring, and support.

*Please see later in the article for the Editors' Summary*

## Abstract 2

Fabienne Shumbusho and colleagues evaluate a task-shifting model of nurse-centered antiretroviral treatment prescribing in rural primary health centers in Rwanda and find that nurses can effectively and safely prescribe ART when given adequate training, mentoring, and support.

## Editors' Summary

**Background:**

Infection with the human immunodeficiency virus (HIV) is a serious health problem in sub-Saharan Africa. The virus attacks white blood cells that protect against infection, most commonly a type of white blood cell called CD4. When a person has been infected with HIV for a long time, the number of CD4 cells they have goes down, resulting in acquired immune deficiency syndrome (AIDS), in which the person's immune system no longer functions effectively.

The World Health Organization (WHO) has divided the disease into four stages as it progresses, according to symptoms including weight loss and so-called opportunistic infections. These are known as clinical stage I, II, III, or IV but were revised and renamed 1, 2, 3, and 4 in September 2005. HIV infection and AIDS cannot be cured but they can be managed with antiretroviral treatment (ART). The WHO currently recommends that ART is begun when the CD4 count falls below 350.

Rwanda is a country situated in the central Africa with a population of around 9 million inhabitants; over 3% of the rural population and 7% of the urban population are infected with HIV. In 2007, the WHO estimated that 220,000 Rwandan children had lost one or both parents to AIDS.

**Why Was This Study Done?:**

The WHO estimates that 9.7 million people with HIV in low- to middle-income countries need ART but at the end of 2007, only 30% of these, including in Rwanda, had access to treatment. In many low-income countries a major factor in this is a lack of doctors. Rwanda, for example, has one doctor per 50,000 inhabitants and one nurse per 3,900 inhabitants.

This situation has led the WHO to recommend “task shifting,” i.e., that the task of prescribing ART should be shifted from doctors to nurses so that more patients can be treated. This type of reorganization is well studied in high-income countries, but the researchers wanted to help develop a system for treating AIDS that would be effective and timely in a predominantly rural, low-income setting such as Rwanda.

**What Did the Researchers Do and Find?:**

In conjunction with the Rwandan Ministry of Health, the researchers developed and piloted a task-shifting program, in which one nurse in each of three rural Rwandan primary health centers (PHCs) was trained to examine HIV patients and prescribe ART in simple cases. Nurses had to complete more than 50 consultations observed by the doctor before being permitted to consult patients independently. More complex cases were referred to a doctor. The authors developed standard checklists, instructions, and evaluation forms to guide nurses and the doctors who supervised them once a week.

The authors evaluated the pilot program by reviewing the records of 1,076 patients who enrolled on it between September 2005 and March 2008. They looked to see whether the nurses had followed guidelines and monitored the patients correctly. They also considered health outcomes for the patients, such as their death rate, their body weight, their CD4 cell count, and whether they maintained contact with caregivers.

They found that by March 2008, 451 patients had been eligible for ART. 435 received treatment and none of the patients were prescribed ART when they should not have been. Only one prescription did not follow national guidelines.

At every visit, nurses were supposed to assess whether patients were taking their drugs and to monitor side effects. They did this and maintained records correctly for the vast majority of the 435 patients who were prescribed ART. 390 patients (over 90%) of the 435 prescribed receiving ART continued to take it and maintain contact with the pilot PHC's program. 29 patients died. Only one was lost to follow up and the others transferred to another ART site. The majority gained weight in the first six months and their CD4 cell counts rose. Outcomes, including death rate, were similar to those treated on the (doctor-led) Rwandan national ART program and other sub-Saharan African national (doctor-led) programs.

**What Do These Findings Mean?:**

The study suggests that nurses are able to prescribe ART safely and effectively in a rural sub-Saharan setting, given sufficient training, mentoring, and support. Nurse-led prescribing of ART could mean that timely, appropriate treatment reaches many more HIV patients. It would reduce the burden of HIV care for doctors, freeing their time for other duties, and the study is already being used by the Rwandan Ministry of Health as a basis for plans to adopt a task-shifting strategy for the national ART program.

The study does have some limitations. The pilot program was funded and designed as a health project to deliver ART in rural areas, rather than a research project to compare nurse-led and doctor-led ART programs. There was no group of equivalent patients treated by doctors rather than nurses for direct comparison, although the authors did compare outcomes with those achieved nationally for doctor-led ART. The most promising sites, nurses, and patients were selected for the pilot and careful monitoring may have been an additional motivation for the nurses and doctors taking part. Health professionals in a scaled-up program may not be as committed as those in the pilot, who were carefully monitored. In addition, the nature of the pilot, which lasted for under three years and recruited new patients throughout, meant that patients were followed up for relatively short periods.

The authors also warn that they did not consider in this study the changes task shifting will make to doctors' roles and the skills required of both doctors and nurses. They recommend that task shifting should be implemented as part of a wider investment in health systems, human resources, training, adapted medical records, tools, and protocols.

**Additional Information:**

Please access these Web sites via the online version of this summary at http://dx.doi.org/10.1371/journal.pmed.1000163.

- PLoS Medicine includes a page collecting together its recent articles on HIV infection and AIDS that includes research articles, perspectives, editorials, and policy forums
- SciDev.net provides news, views, and information about science, technology, and the developing world, including a section specific to HIV/AIDs
- The World Health Organization (WHO) has published a downloadable booklet Task Shifting to Tackle Health Worker Shortages
- The WHO offers information on HIV and AIDS (in Arabic, Chinese, English, French, Russian, and Spanish) as well as health information and fact sheets on individual countries, including on Rwanda
- The UNAIDS/WHO working group on HIV/AIDS and Sexually Transmitted Infections (STI) Surveillance gathers and publishes data on the prevalence of HIV and AIDS in individual countries, including on Rwanda
- AIDS.ORG provides information to help prevent HIV infections and to improve the lives of those affected by HIV and AIDS. Factsheets on many aspects of HIV and AIDS are available. It is the official online publisher of AIDS Treatment News

## Background

Although significant progress has been made in the provision of antiretroviral treatment (ART) in sub-Saharan Africa, only 30% of patients eligible for ART were receiving treatment by the end of 2007 [1]. In many countries, the shortage of human resources for health is one of the major barriers to achieve universal access to HIV care and treatment [2]. In particular, reliance on physician- and hospital-centered care hampers the ability to scale-up ART. As a result, the World Health Organization (WHO) has recommended task shifting—the process of delegation of tasks to health workers with lower qualifications—as a necessary and critical dimension of decentralized AIDS care in Africa [3],[4]. Whereas task shifting, including that for HIV care delivery, is well-studied and established in rich countries, only limited published data exist from HIV settings in sub-Saharan Africa [5]. As such, the level of training and support needed to implement task-shifting models, and the quality of care provided within these settings, remains largely unknown. Consequently, national ART programs in resource-limited countries have been slow to adopt this approach.

With a population of around 9 million inhabitants, Rwanda has an overall HIV prevalence of 3% and more than 7% in urban areas [6]. The national ART program, launched in 2003, was first established at the district level and was subsequently decentralized to primary health centers (PHCs); by the end of 2007, 171 (39%) of the 434 health facilities provided ART services, most of these being PHCs [7]. The Government of Rwanda currently does not allow ART prescription by nurses. However, with one physician per 50,000 inhabitants and one nurse per 3,900 habitants, Rwanda faces a severe physician shortage but is relatively better resourced with nurses [8]. In addition, 80%–90% of the population is rural-based and relies primarily on health services at PHCs staffed by nurses. Even at the PHC level, the bulk of HIV care is medical doctor (MD)-centered and provided by visiting physicians, typically based at district hospitals. With the permission of the Rwanda Ministry of Health (MOH), in 2005 Family Health International (FHI) developed a task-shifting model to assess the feasibility and effectiveness of nurse-initiated AIDS treatment as a basis for further national policy development. In three rural PHCs, nurses were trained to prescribe first-line ART regimens for treatment-naïve adult patients with ongoing physician supervision and mentoring.

In this article, we report evaluation results of the pilot intervention. Specifically, we present descriptive data extracted from patient records to assess (i) nurse performance vis-à-vis compliance with national clinical guidelines for HIV care and treatment, and (ii) key patient outcomes, including retention, body weight, and CD4 cell count change after treatment initiation. The initiative was funded as an intervention rather than as a research project, and limited resources precluded using a comparative study design. However, we hope that these data will help to inform future efforts to either scale-up the current initiative in Rwanda or plan similar initiatives elsewhere.

## Methods

### ART Patient Management

The intervention was designed to enable nurses at PHCs within existing ART services to prescribe first-line ART regimens, ensure the management of ART-naïve adult cases, and organize referral of complex cases. Pediatric patients, ART non-naïve adults, and patients with associated medical conditions (e.g., tuberculosis), or severe baseline laboratory abnormalities, were considered complex and were excluded from nurse-initiated treatment. These patients were referred to the supervising physician for ART initiation, follow-up, or both. Table 1 summarizes the differences between this nurse-centered and the traditional physician-centered models of AIDS care.

**Table 1:** Role of physicians and nurses in the traditional MD-centered HIV/ART care program and the nurse-centered pilot project.

Phase of Care	Tasks	MD-Centered		Nurse-Centered	
		Role MD	Role Nurse	Role MD	Role Nurse
**Pre-ART care**	**Initial physical exam/staging**	X	—	—	X
	**Ordering CD4 count**	X	X	—	X
	**Assessment of ART eligibility**	X	—	—	X
	**Follow-up of noneligible patients**	X	—	—	X
	**Cotrimoxazole refill**	—	X	—	X^a^
	**Complex medical cases** b	X	—	X	—
**ART care**	**Ordering lab tests**	X	X	—	X
	**Interpretation of lab tests**	X	—	—	X
	**ART initiation and FU of noncomplex cases**	X	—	—	X
	**ART initiation and FU of complex cases** b	X	—	X	—
	**ART refill**	—	X	—	X^a^
	**Register keeping/reporting**	—	X	—	X
	**Filing of results/medical records**	—	X	—	X^a^
	**Training/mentoring**	—	—	X	—
	**Supervision**	—	—	X	—

a These tasks were taken over from the prescribing nurse by other staff (nurses, social workers).

b Complex cases included those with advanced HIV disease, severe/persistent opportunistic infections, severe ART side effects, suspicion of ART failure, severe or recurrent nonadherence to ART, pediatric (<15 y), and ART-experienced patients.

Patients were started on ART according to the national guidelines, which are based on WHO clinical and immunological eligibility criteria: WHO clinical stage IV without consideration of CD4 count; CD4 count ≤200/µl with previous guidelines and ≤350/µl with revised July 2007 guidelines. A majority of patients received a generic, fixed-dose combination of stavudine (d4T), lamivudine (3TC), and nevirapine (NVP). From the beginning of 2008, zidovudine (AZT) replaced d4T in the preferred first-line regimen in the national guidelines. Nurses in this pilot program were instructed about contraindications to specific ARV medications per national preservice training. For those with baseline neuropathy, d4T was to be replaced with AZT, except for patients with concurrent anemia (hemoglobin <9 g/dl). NVP was replaced by efavirenz (EFV) for tuberculosis patients or those with grade III/IV liver toxicity at baseline, after excluding pregnancy.

ART adherence was evaluated at each patient visit by self-report and pill counts. Side effects were evaluated at each follow-up visit, and treatment changes were made for grade 3 and 4 events [9]. For those cases, changes of treatment were made by the visiting MD. Prior to a change in treatment, interruptions to treatment might have been indicated for various reasons, such as an allergic reaction; if such cases occurred when no MD was on hand and if no transfer to the hospital was organized, the nurse made the interruption. Liver function tests were performed at baseline, 2, 4, and 8 wk, and thereafter every 6 mo and on clinical grounds. A full blood count (FBC) was done at baseline, 12 wk, and thereafter every 6 mo and on clinical grounds. For patients on AZT, an additional FBC control was done at 4 and 8 wk. From the beginning of 2007, laboratory monitoring was simplified according to the national guidelines, with measurement of serum glucose, amylase, transaminases, and creatinine for cases presenting indications only but not for routine exams, and keeping hemogram as an initial mandatory exam and on a regular basis in case of AZT. CD4 cell count measurements, checked every 6 mo while on ART, were performed at the laboratory of the District Hospital using FACSCount technology (Becton-Dickenson).

### Study Setting and Description of the Intervention

In consultation with the MOH, FHI provided technical assistance to four rural PHCs to implement the intervention. Selection criteria included: (i) comprehensive services for HIV care in place (counselling and testing, prevention of mother-to-child transmission, and AIDS patient care and treatment); (ii) supervision and support by a district hospital that was already providing ART to patients; and (iii) a nurse on staff who had at least 2 y experience providing clinical care for non-HIV pathologies. Other factors for PHC selection were geographic diversity, infrastructure, and overall staffing patterns that were considered standard in Rwanda. Of the four PHCs initially selected, one was later excluded because of staffing changes that precluded nurse-initiated ART. Data from this PHC are not included in the current analysis. Characteristics of the three remaining PHCs are similar to other rural sites in Rwanda, with catchment area populations of 15,000 to 28,000 inhabitants, 45 to 117 km from the capital Kigali, none connected to the electric grid, and ten to 17 staff, with some of those being additional personnel provided for this intervention. Estimated HIV prevalence within the two districts where the three PHCs are located was 5.3% (Muhanga) and 4.6% (Nyanza), based on 2007 antenatal consultation (ANC) sentinel surveillance.

One nurse at each PHC was trained by a physician from FHI to: (i) perform physical exams of HIV patients and to order and interpret blood tests during patient enrollment and follow-up; (ii) prescribe ART to noncomplex adult cases; and (iii) refer complex cases, including children, to the physician (MD) (Table 1). Selected nurses first received formal training in national treatment guidelines, followed by a 5-d practicum training at an established ART site and 5–10 d “bedside” training (see Table 2). “Bedside” training involved nurse-consultation of all HIV-positive patients (ART-eligible and noneligible) under the observation of the physician. After a minimum of 50 physician-observed consultations had been completed with ART-eligible patients, nurses were allowed to consult patients independently but were provided ongoing physician support from a distance with weekly supervision and mentoring at the PHC treatment site. In each facility, an additional nurse received the training, in order to ensure replacement of staff in the ART service in case of need.

**Table 2:** Training and supervision activities within the pilot project.

Activity	Description
Training	
Theoretical training	2 wk-training on HIV care and ART (as part of national training program organized for physicians, nurses, and social workers)
Practical training	5 d-training in established ART site (as part of national training program): introduction to nursing aspects of ART including pre-ART patient preparation and adherence support, dispensing ARVs, organizational and reporting activities
On-the-job training	“Bed-side teaching”- training with the FHI MD at the pilot site (5–10 d in total): joint consultation with emphasis on ARVs, monitoring (side effects, adherence, laboratory); clinical skills; OI diagnosis and management. Supervised prescription of ART (once weekly visits); minimum 50 cases initiated and monitored under supervision. Supervision tool for evaluation. Telephonic consult/advice by MD at all times. Formal evaluation prior to prescribing ART independently.
Supervision	
By District MD^a^	Weekly visits to confirm ART prescriptions, review medical/complex cases and supervise monitoring activities; ongoing training and mentoring
By FHI MD	Supervision of activities of ART team (nurse and district MD) on: medical aspects (case discussions, patient file review); completeness of patient files and program registers. Monthly visits at start, later every 3 mo. Ongoing training and mentoring. Guided by supervision tool.

a Given the shortage of MDs at one district hospital, the FHI MD temporarily functioned as the district MD at two intervention sites.

OI, opportunistic infections.

While the nurses participating in the pilot intervention were specifically assigned to attend to HIV patients, they continued to play a role in overall patient care according to each facility's organization of clinic staff. The nurses did not receive salary increases or other incentives for their new role under this task-shifting initiative.

MDs made weekly visits to the intervention PHCs. As indicated in Table 1, during these visits, MDs ensured the management of complex cases, including cases of advanced HIV disease, severe opportunistic infections, severe ART side effects, suspicion of ART failure, severe or recurrent nonadherence to ART, and ART-experienced patients. MDs also verified the prescriptions made by nurses, and provided guidance and advice on specific cases, general aspects of care, and the organization of services (Table 2).

To facilitate nurse-initiated ART prescription and patient management, the national patient record, designed for use by MDs, was adapted for use by nurses following WHO task-shifting recommendations [10]. We developed check-lists to guide nurses to collect and assess essential data elements including: sociodemographic characteristics of the patient; baseline clinical assessment and WHO staging; contraindications for nurse-ART prescription; and screening for side effects and nonadherence during follow-up appointments. To clarify and reinforce nurse responsibilities and indications for physician referral, we developed an algorithm for patient management. Finally, we used standardized evaluation forms to guide routine MD supervision.

### Data Collection and Statistical Analysis

Patient records were examined retrospectively to assess nurse performance in implementing national treatment guidelines and patient outcomes under nurse care. All adult patients who were enrolled into HIV care or who started ART after the pilot start-up in September 2005 and up to the time of data abstraction in March 2008 were included in the assessment. Patients who were transferred from other sites while on ART or who started prophylactic ART for prevention of mother-to-children transmission were excluded from assessment.

Completeness of patient data was assessed at baseline for the following parameters: WHO clinical stage, functional status [10], weight, review of systems, and physical exam; baseline CD4 cell count and laboratory tests; screening for tuberculosis and reproductive health issues. ART eligibility was ascertained and compared with the nurse's decision. For patients determined eligible, the presence of contraindications for nurse ART prescription was assessed. For patients started on ART, the correctness of the ART prescription was verified as per the national guideline (combination of drugs, prescription despite contraindication). The completeness of follow-up was assessed for weight and CD4 cell count measurements, adherence assessments, and screening for side effects. To assess the nurse's responsiveness towards persistent side effects, defined as those reported at three or more consecutive follow-up visits, we evaluated whether the side effect management was documented and whether the patient had been appropriately referred to the physician.

The key patient outcomes assessed were patient retention by the time of data abstraction, CD4 cell count, and weight evolution after ART initiation. Clinical status was defined as: alive and on ART, lost to follow-up (LTFU; LTFU was defined as “not showing or absent at the health center for more than 3 mo since the last visit, without alternative explanation”), transferred to another site, or died. No data on the cause of death were collected. Since varying definitions of retention can be found in the literature, we used the two most commonly reported definitions. Under the first definition, patients are considered to be “retained in the system” if they are known to be alive and on ART at the time of assessment, including transfers to other ART sites [11]. Under the second definition, patients who transferred to another ART site are considered to be nonretained (“retention at the facility level”). The proportion of patients requiring toxicity-related drug substitution and regimen switch for treatment failure was reported as well.

Data were collected from the three sites by a team consisting of three trained data entry persons and one supervisor. Data were abstracted from the patient files and entered into an Access-database using a data-entry mask in Epi Info (version 3.3.2, US Centers for Disease Control).

For mortality and retention, we estimated cumulative event probabilities through 24 mo after ART initiation using Kaplan-Meier methods. For mortality, estimated curves were stratified by baseline WHO stage and CD4 cell count and we used the log-rank test for comparing groups.

For describing CD4 cell count and weight evolution over time following ART initiation, we applied general linear mixed models using restricted maximum likelihood. To avoid a false perception of improving outcomes caused by sicker patients dying early, we excluded from the analyses of CD4 and weight all patients who died, were lost, or were transferred prior to their 6-mo assessment. Models included fixed effects for baseline characteristics, including health center, WHO stage, age, gender, and ART regimen. Additionally, the CD4 model included a term for baseline weight and the weight model included a term for baseline CD4. We also included as fixed effects polynomials of time and interactions between WHO stage and time. For CD4, a quadratic model fit adequately (based on a likelihood ratio test), while for weight cubic terms were required. Random effects included patient-specific intercept, slope, and quadratic term increments. Change over time was assessed using appropriately specified contrasts. Model and test results are presented for the purposes of describing our study population only and are not intended for making inferences to any larger population. All analyses were performed using SAS, Version 9.1 (SAS Institute).

### Ethical Considerations

This study analyzed routine data of the HIV care program. The Rwandan National Ethics Committee (RNEC, Kigali, Rwanda) gave exemption from formal ethical review.

## Results

Between September 2005 and March 2008, 1,076 adult patients were enrolled into HIV care or started lifelong ART within the pilot program. Of these, 710 (66%) were women and the median age was 38 y (interquartile range [IQR] 31–45) (Table 3). Most were WHO clinical stage I (53%) or WHO clinical stage II (28%) at the time of enrollment. Overall, 435 patients (40%) were started on ART, with a median baseline CD4 cell count of 184 cells/µl (IQR 116–231). Most patients who initiated ART were either WHO clinical stage II (39%) or III (35%); only 2% were stage IV. The median follow-up time on ART at the time of assessment was 8.3 mo (IQR 2.6–15.9).

**Table 3:** Demographic and clinical characteristics of patients enrolled into the pilot study.

Characteristic	Pre-ART (*n* = 641)	ART (*n* = 435)	Total (*n* = 1,076)
**Female**	433 (67.7%)	276 (63.4%)	710 (66.0)
**Age (y)**	37 (30–44)	39 (33–46)	38 (31–45)
**WHO clinical stage**			
**Stage I**	462 (72.4%)	103 (23.7%)	565 (52.7%)
**Stage II**	127 (19.9%)	171 (39.3%)	298 (27.8%)
**Stage III**	47 (7.4%)	152 (34.9%)	199 (18.5%)
**Stage IV**	2 (0.3%)	9 (2.1%)	11 (1.0%)
**Baseline weight (kg)**	54 (49–59)	52 (46–58)	53 (46–58)
**Baseline CD4 count (cells/µl)**	643 (464–846)	184 (116-–231)	382 (194–694)
**Time since enrolment (mo)**	4.1 (0.6–11.7)	14.4 (7.7–22.2)	8.0 (1.7–16.7)
**Time on ART (mo)**	—	8.3 (2.6–15.9)	—

Data reported as either frequency (percent) or as median (IQR).

Of the 622 patients who were determined to be ineligible for ART, none were started on treatment. We identified 451 patients who were eligible for ART, of which 435 (96%) started treatment. The other 16 (4%) did not start treatment for various reasons: six did not return for ART initiation, five were receiving pre-ART counselling at the time of data collection, one was transferred after the initial assessment, and two died prior to ART initiation. For two cases, we did not find a clear explanation for the delay in ART initiation. The majority of patients were reported to be screened for tuberculosis, had reproductive health issues assessed, were clinically examined, and were assessed for contraindications for nurse-initiated treatment (Table 4). For those who initiated ART, the median time between the HIV test and ART initiation was 27 d (IQR 16–42), with a median of 15 d (IQR 10–24) between CD4 sample collection and ART initiation.

**Table 4:** Correctness of eligibility assessment and completeness of patient record at intake and during follow-up of patients started on antiretroviral treatment (*n* = 1,076).

Indicator^a^	*n* (%)
Assessment at intake among patients initiating ART (*n* = 435)	
Physical examination complete	422 (97.0)
Tuberculosis screening complete (four questions)	426 (97.9)
Reproductive health assessment complete (*n* = 276)^b^	232 (84.0)
All ART contraindications evaluated	430 (98.8)
WHO clinical staging documented	435 (100)
Functional status documented	426 (97.9)
Baseline CD4 count documented	434 (99.8)
Baseline weight documented	434 (99.8)
Baseline laboratory tests documented	406 (93.3)
Eligibility assessment^c^	
Ineligible patients started on ART (*n* = 622)	0 (0)
Eligible patients not started on ART (*n* = 451)	16 (3.5)
Assessment during follow-up	
Side effect screening at every visit (*n* = 386)^d^	322 (83.4)
Adherence assessed at every visit (*n* = 240)^d^^,^ e	214 (89.2)
CD4 count/weight available^f^	
At 6 mo (*n* = 217)	193 (88.9)/200 (92.2)
At 12 mo (*n* = 123)	104 (84.5)/111 (90.2)
At 18 mo (*n* = 43)	31 (72.1)/28 (65.1)
At 24 mo (*n* = 10)	10 (100.0)/6 (60.0)

a *n* = 435 unless stated otherwise.

b Only for female patients (date of last menstrual periods, contraceptive use; pregnancy and breastfeeding status); considered complete if all questions adequately addressed.

c Eligibility could not be determined for three patients since baseline WHO stage or CD4 count not documented.

d Only considering patients starting ART at least 1 mo prior to data collection.

e Excluding one site where data on adherence were recorded differently.

f Within 3 mo of the time point.

The most commonly prescribed regimens were d4T+3TC+NVP (69%) and AZT+3TC+NVP (26%); d4T+3TC+EFV (4%) and AZT+3TC+EFV (1%) were used less frequently. All drug prescriptions were consistent with the national guidelines, except that one patient was given EFV without prior exclusion of pregnancy. In no observed cases did nurses initiate ART in the presence of a contraindication. Twenty-three (5.3%) patients changed their initial ART regimen and, of these, the majority were single-drug substitutions due to toxicity. Only three patients were started on second-line ART for treatment failure by the visiting MDs. During follow-up, ART adherence, based on self-report, was assessed at every visit for 89% of the patients, and screening for side effects was performed regularly for 83%. For the seven patients documented to have persistent side effects, management was clearly documented for five (71%). CD4 count results and weight measurements while on treatment were available for the majority of patients (Table 4).

**Figure 1:**
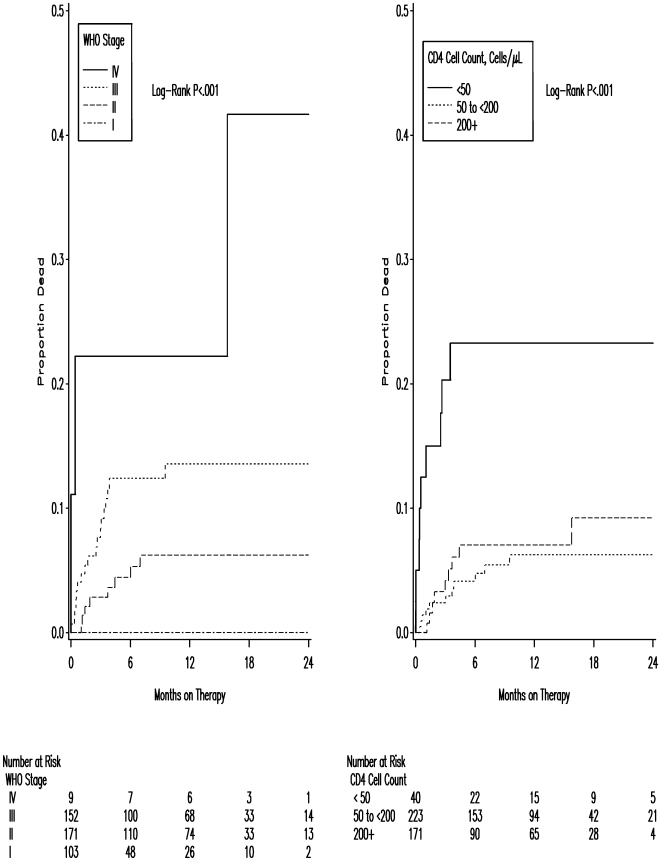
Kaplan-Meier plots showing mortality of 435 patients after initiation of ART by baseline WHO clinical stage (left) and CD4 cell count (right).

By the time of assessment, 390 (90%) of the 435 patients initiating ART were alive and on ART within the pilot PHCs. Twenty-nine (7%) patients had died, one (<1%) was LFTU, and 15 (3%) had been transferred to another ART site. The median time on treatment for those who died was 1.9 mo (IQR 0.7–3.7); 62% of deaths occurred within 3 mo after treatment initiation, and 86% within 6 mo. As seen in Figure 1, mortality was strongly associated with advanced WHO disease stage and low baseline CD4 count.

**Figure 2:**
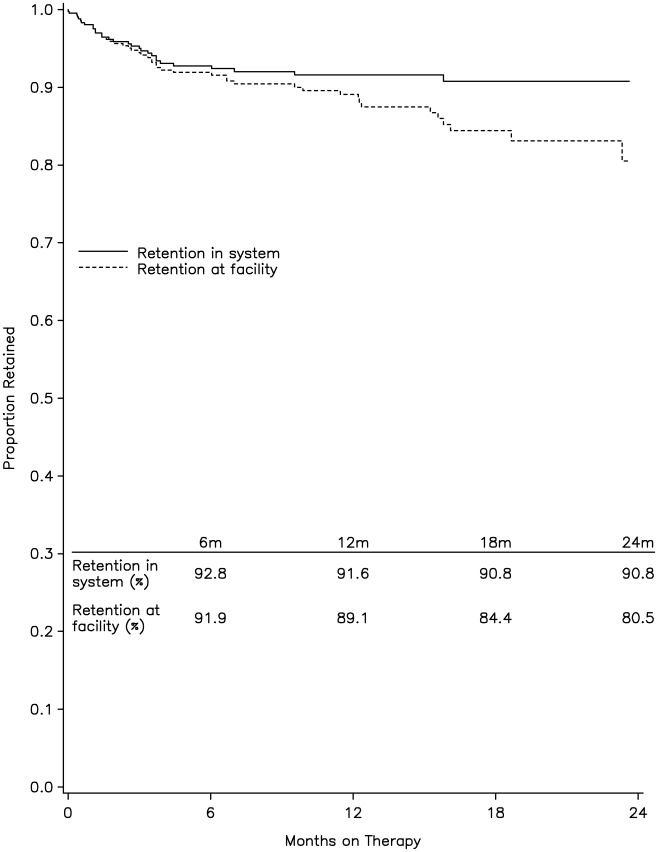
Kaplan-Meier plot showing retention into care for the 435 patients started on treatment.

Estimated retention in the system was 93% at 6 mo, 92% at 12 mo, and 91% at 18 and 24 mo (Figure 2). Estimated retention at the facility level decreased to 80% by 24 mo. The model-estimated mean CD4 cell count and weight changes while on treatment, stratified by WHO stage, are summarized in Table 5. Depending on initial WHO stage, increases in mean CD4 cell count ranged from 97 to 128 cells/µl from pre-ART to 6 mo following treatment initiation and from 79 to 129 cells/µl from 6 to 24 mo after treatment initiation. Similarly, mean weight significantly increased between 1.8 and 4.3 kg in the first 6 mo following treatment initiation; mean weight also increased between 0.5 and 1.7 kg in the period between 6 and 24 mo, however these increases were not significantly different from zero.

**Table 5:** Model-estimated mean change in CD4 cell count and weight after initiation of ART, by baseline WHO stage (*n* = 404).

Measures of clinical and Biological Status	Between 0 to 6 mo Mean (95% CI)	Between 6 to 24 mo Mean (95% CI)
**Weight change (kg)**		
**WHO Stage I**	1.8 (0.6–3.0)	0.5 (−3.8 to 4.7)
**WHO Stage II**	1.8 (1.0–2.6)	1.0 (−1.3 to 3.3)
**WHO Stage III/IV**	4.4 (3.5–5.2)	1.8 (−0.2 to 3.7)
**CD4 cell count (cells/µl)**		
**WHO Stage I**	96.7 (69.4–124.1)	126.0 (−9.6 to 261.5)
**WHO Stage II**	94.0 (76.1–111.7)	79.1 (14.2–144.0)
**WHO Stage III/IV**	128.0 (108.7–147.3)	128.9 (65.0–192.7)

Models also control for site, age, gender, and ART regimen (AZT-containing and/or NVP-containing). CD4 model also controls for baseline weight, dichotomized at the gender-specific median. Excludes 30 patients who either died or were transferred prior to the 6-mo visit/measurement.

## Discussion

To our knowledge, this is the first assessment of nurse-centered ART within a context of a nationally coordinated program seeking to achieve universal access to AIDS care and treatment. Our evaluation suggests that with adequate preparation and support nurses can effectively prescribe first-line ART and monitor noncomplex patients. Nurses participating in the initiative achieved high compliance with national guidelines and excellent patient outcomes. The experience demonstrates the feasibility and suggests effectiveness of nurse-centered task shifting for decentralized ART services without compromising the quality of care.

Our findings bear out positive reports from other African countries. While Morris et al. [12] describe a task-shifting strategy used in clinics in Lusaka, Zambia, Stringer and colleagues [13] report robust treatment adherence and patient outcomes from a large cohort at urban primary care sites in the city. The treatment services relied primarily on nonphysician clinicians to provide initial patient management with rotating physicians consulting complex patients. Specialist HIV clinicians outside of Zambia could be consulted as well via internet [12]. While confirming the feasibility of involving nonphysician clinicians, the Zambian program relied heavily on clinical officers, who have received substantial medical training but are of limited availability in many African countries. In addition, no formal assessment of the performance of the nonphysician clinicians was reported and no details were provided on the practical organization (training, task-distribution), implementation, and supervision within the program.

In South Africa, an expanded role for nurses in AIDS care, including nurse-initiated ART, was tried in order to enhance service delivery in rural communities [14]. An assessment of program data from Lusikisiki showed significantly increased enrollment of patients on treatment through decentralized rural services, whereas the hospital ART enrollment capacity was rapidly exceeded. Furthermore, a comparison of 1-y outcome data on retention, CD4 monitoring and response, and viral load consistently showed outcomes for rural clinic patients to be significantly better than patients treated at hospitals. No assessment of nurses' performance was reported, however, and details of the step-by-step organization of nurse-initiated ART was lacking.

That the task-shifting initiative in Rwanda was carried out within a national framework with the explicit objective to evaluate its safety and potential for policy development represents an important addition to the existing literature. Our report provides a detailed description of the structure and content of the intervention as well as additional evidence that nurse-initiated ART may be a viable strategy for expanding access to AIDS treatment to rural populations. In combination with an independent evaluation commissioned by the Ministry of Health in early 2008 [15], the present evaluation forms the basis of national plans to adopt this task-shifting strategy for national-level ART scale-up.

Compared to a recent evaluation of the national ART program in Rwanda during 2004–2005, our study found that patient retention at the facility level was 89% at 12 mo of ART, close to the national estimate of 87% for adult patients attending similar size health centers in Rwanda, where care is generally provided by physicians [16]. Whereas documented mortality at 12 mo was slightly higher in our program (8.5% versus 5.7%), LTFU was substantially lower (0.3% versus 3.2%). Comparable changes in weight and CD4 cell count were also observed. Overall, key patient outcomes from this task-shifting model were also comparable to findings from other cohorts in sub-Saharan Africa. For example, in a recent systematic review of ART programs in sub-Saharan Africa, retention at 1 y after ART initiation was estimated at 70% [11]. Similar mortality rates, stratified by baseline CD4 count or WHO stage, were reported in large programs in Zambia [13], Malawi [17], and numerous other countries in the region [16],[18].

The limitations of this study include the fact that data presented are descriptive and no direct comparison of outcomes within this nurse-centered model of care was made with those from traditional physician-centered models, which makes it difficult to ascertain if patients' outcomes were as a result of the nurses' role or due to MDs' intensive supervision. Furthermore, we should exercise caution when interpreting the retention results given that the median follow-up time for patients in this sample was 8.3 mo. Although the sites selected were generally comparable to other PHCs in Rwanda, the fact that neither the sites nor the patients were randomly selected implies that our findings cannot necessarily be generalized to other sites or even to other patients at these particular sites. The criteria used to identify pilot sites favored PHCs that offered relatively favorable conditions, particularly with strong management and adequate staffing. Also, we selected nurses with substantial clinical experience prior to the pilot initiative. Finally, participation in a closely monitored pilot project can itself increase commitment from both nurses and physicians. Future sites will need to meet a minimum set of criteria for safe and effective nurse-initiated ART delivery.

To avoid over-reliance on nursing skills for HIV care and treatment, the tasks and responsibilities of nurses and physicians will also need to be clear. As we task shift some clinical responsibilities to nurses, we must also consider the modified role of physicians, which will require provision of care for complex patients while at the same time providing overall oversight of patient management by the nurses. Not only provider roles but skill levels (of both nurses and physicians) will need to be reviewed, periodically revised, and upgraded to meet evolving clinical challenges. In particular, treatment failure will be an important challenge in the future, as will provision of treatment and follow-up to children. Both will require careful monitoring and an appropriate mix of physician and nurse skills. To emphasize the complementary nurse-physician skill mix necessary to provide high quality patient care, it might be more appropriate to conceptualize this approach as “task sharing” rather than task shifting. Specific guidelines to scale-up the initiative should thus focus on defining roles and responsibilities of the nurse-physician patient care team. Additionally, this nurse-physician task shifting is best framed within a whole context of task transfers for patient care, including to community health workers, family members, and patients themselves.

In addition, regulatory frameworks integrating task shifting within national policy on human resources for health should be established [19],[20]. Given the severe shortage of human resources for health, task shifting in isolation is not recommended but rather as combined with wider investment in human resources and health systems strengthening more generally [20]. High quality preservice training with regular in-service updates, focusing not only on theory but actual clinical practice, will be pivotal for the long-term success of task shifting [20]; training curricula will need to be tailored to different nursing and nonphysician practitioners providing care in different African settings [21]. Adapted medical records, tools, and protocols to assist nurses and other health professionals to perform in this expanded AIDS care role will also be necessary [21]. Further research on successful and cost-effective implementation models, as well as studies on patient and health care worker perspectives will also be of interest [5].

The potential benefit of this task-shifting model likely extends beyond HIV patient care, as indicated by Chung and colleagues [22]. Including time estimates for physician supervision and consultation in complicated cases, Chung and colleagues estimate that nurse-initiated ART for noncomplex cases saved approximately 45 min of physician time for every 1 h worked by a prescribing nurse. If task shifting were applied nationally to the future roll-out of ART in Rwanda, their simulation model suggested a 76% reduction in the HIV care burden for physicians. Physician time spared through ART task shifting could be used for other patient care, mentoring, and quality improvement activities. On this point, specific guidance and systems may be in order; whereas the task of the nurses was clearly defined and evaluated in this pilot initiative, the new roles that physicians could play was not explored. On the other hand, the impact of this model on the overall health system, and specifically on other activities where the nurses would have been used, was not explored either.

Given the shortage of physicians in many African countries [8], nurses have long played an important role in the management and treatment of key diseases, such as tuberculosis and malaria, typically with well-established task descriptions, clear referral indications, and documented good results [5]. This report adds to the evidence base that nurses can play a similarly effective role in HIV care. It further advocates for decentralized HIV care and treatment at the primary health clinic level [23]. If carefully conceived and monitored, task shifting for HIV care offers a promising strategy to address physician shortages in many resource-limited countries and will likely have benefits across the health sector.

## Abbreviations

3TC: lamivudine
ART: antiretroviral treatment
AZT: zidovudine
d4T: stavudine
EFV: efavirenz
FHI: Family Health International
IQR: interquartile range
LTFU: lost to follow-up
MD: medical doctor
NVP: nevirapine
PHC: primary health center
